# Establishment of the mid-sagittal reference plane for three-dimensional assessment of facial asymmetry: a systematic review: Establishment of the mid-sagittal reference plane: a systematic review

**DOI:** 10.1007/s00784-024-05620-7

**Published:** 2024-04-05

**Authors:** Deepal Haresh Ajmera, Pradeep Singh, Yiu Yan Leung, Balvinder S. Khambay, Min Gu

**Affiliations:** 1 Discipline of Orthodontics, Faculty of Dentistry, The University of Hong Kong, Hong Kong SAR, China; 2 Discipline of Oral and Maxillofacial Surgery, Faculty of Dentistry, The University of Hong Kong, Hong Kong SAR, China; 3 Orthodontics Department, School of Dentistry, University of Birmingham, Birmingham, UK; 4 Discipline of Orthodontics, Faculty of Dentistry, the University of Hong Kong, Hong Kong SAR, China

**Keywords:** Facial asymmetry, 3D, Three-dimensional, MSP, Mid-sagittal reference plane, Orthognathic surgery

## Abstract

**Objective:**

To systematically review the literature for mid-sagittal plane establishment approaches to identify the most effective method for constructing the mid-sagittal plane for the evaluation of facial asymmetry.

**Materials and methods:**

Six electronic databases (PubMed, Medline (via Ovid), EMBASE (via Ovid), Cochrane Library, Web of Science, and Scopus) and grey literature were searched for the studies that computed the mid-sagittal reference plane three-dimensionally, using a combination of MeSH terms and keywords. The methodological quality and the level of evidence for the included studies were analyzed using QUADAS-2 and GRADE, respectively.

**Results:**

The preliminary search yielded 6746 records, of which 42 articles that met the predefined inclusion criteria were included in the final analysis. All the included articles reported the construction of the mid-sagittal reference plane (MSP) using varied methods. The risk of bias and concerns regarding the applicability of the included studies were judged to be ‘low’. The level of evidence was determined to be ‘low’ for the effectiveness of the technique and ‘moderate’ for the ease of clinical applicability.

**Conclusion:**

Despite methodological heterogeneity, this review substantiates the comparable efficacy of cephalometric and morphometric MSP construction methods. A fully automated morphometric MSP holds promise as a viable option for routine clinical use. Nevertheless, future prospective studies with an emphasis on the impact, accuracy, and clinical applicability of MSP construction techniques in cases of facial asymmetry are required.

**Clinical relevance:**

The present review will assist clinicians in selecting the most suitable method for MSP construction, leading to improved treatment planning and ultimately more favorable treatment outcomes.

**Supplementary information:**

The online version contains supplementary material available at 10.1007/s00784-024-05620-7.

## Introduction

Facial asymmetry has a markedly detrimental psychosocial, aesthetic, and functional effect on an individual’s quality of life [1]. Consequently, patients with true skeletal facial asymmetry often seek surgical correction [2]. This is commonly achieved by complex three-dimensional (3D) surgical movements aimed at restoring their facial symmetry in line with their peers. The key to an aesthetic symmetrical outcome relies on determining the site and severity of the facial disharmony, which in turn is determined by the accuracy of the estimated sagittal plane.

Two-dimensional (2D) postero-anterior cephalography and full-face photographs [3, 4] have historically been used to evaluate facial asymmetry. However, the information provided is limited because they cannot accurately depict the true three-dimensional nature of the facial skeleton [5, 6]. The advent of 3D imaging and its routine use in various fields, such as orthodontics and oral and maxillofacial surgery, has revolutionized the process of diagnosing, planning, and predicting the outcomes of surgery [7–10]. Regardless of the imaging modality, determining the mid-sagittal plane (MSP) is fundamental to the process [4, 11–13]. Several approaches for establishing a plane of symmetry have been used, depending on the image type being analysed. These have included simple landmarks for 2D cephalograms and form, shape, or landmark configuration for 3D images. In addition, an array of nomenclature has been documented for the plane of symmetry [1, 3, 14, 15]. These include ‘Best Symmetry Plane [16], Symmetry Plane, or Primal Sagittal Plane [17]. However, there is no agreement as to which method defines a clinically valid MSP in patients presenting with facial disharmony [15], and to date, no paper has systematically reviewed all the published methods that are currently available. Therefore, the aim of this paper was to systematically review the literature for different approaches utilized to establish the mid-sagittal plane in order to choose the most effective method for constructing the mid-sagittal plane for the evaluation of facial asymmetry.

## Materials and methods

### Protocol & registration

The systematic review reported follows the *PRISMA guidelines: Preferred Reporting Items for Systematic Reviews and Meta-Analyses* [18]. The review protocol was registered with the PROSPERO database (registration number: CRD42020218963; https://www.crd.york.ac.uk/prospero/display_record.php?ID=CRD42020218963).

### Eligibility criteria

The following focused topic that matched the *Population-Intervention-Control-Outcome (PICO)* criteria was the subject of a literature search: “What is the best technique to construct a mid-sagittal reference plane for the estimation of facial asymmetry?” For this review, studies that addressed the aforementioned question were judged appropriate. The elements for PICO criteria have been listed in Table 1. Studies were considered if they met the following inclusion criteria: 1) utilized a 3D technology-based tool, device, or software or conducted any intervention for the estimation of MSP; 2) conducted on humans and had the proper analytical design, such as case–control studies, cross-sectional studies, prospective studies, and retrospective studies, including pilot studies; 3) study data did not duplicate or overlap with those of other articles; 4) studies had full text availability and were published in English. Studies performed on animals, non-human models, non-pertinent data, not involving facial asymmetry subjects or focused on 2D analysis, letters to editors, conference papers, and review articles were excluded from the current systematic review.

**Table 1:** Description of the PICO (P = population; I = intervention; C = comparator/control O = outcomes) elements used in structuring the research question and the search strategy

Criteria	Specification
Focus question	What is the best technique to construct a mid-sagittal reference plane for the estimation of facial asymmetry?
Population	Patients with clinically diagnosed facial asymmetry or craniofacial deformity
Intervention	The use of any three-dimensional (3D) technology-based tool, device, software, or intervention for the estimation of the mid-sagittal plane
Comparator/control	Different midsagittal plane construction methods; different types of midsagittal planes; asymmetry or normal controls; different asymmetry quantification methods
Outcomes	a) Effectiveness of the technique b) Ease of clinical applicability
Search strategy	Search (3-dimensional OR 3-D OR 3D OR three-dimensional OR mesh (three-dimensional)) AND (midsagittal OR midsagittal OR (midsagittal plane) OR MSP OR MRP OR (midsagittal reference plane)) AND ((facial asymmetry) OR (asymmetric face) OR (asymmetrical face) OR mesh (facial asymmetry))

### Information sources and literature search

All the relevant studies were identified systematically and independently by two authors (DA and PS) through a comprehensive search in the electronic databases:*PubMed, EMBASE (*via* Ovid), Medline (*via* Ovid), Cochrane Library, Scopus, and Web of Science* (until June 2023) with the combination of Medical Subject Headings (MeSH) terms as keywords. Moreover, vocabulary and syntax were adjusted across the databases. The literature search was not constrained by publication date or status. In addition, manual and OpenGrey database (http://www.opengrey.eu/) searches were carried out.

### Study selection

After conducting a comprehensive literature search, two authors (DA and PS) independently conducted an initial evaluation of the titles and abstracts of potential to ensure their eligibility according to the predetermined inclusion and exclusion criteria. Disagreements over the inclusion of the studies at this stage were solved by discussion. Next, the full-text studies that conformed to the inclusion criteria were retrieved. The Cohen’s kappa statistic (κ) was used to determine the inter-reviewer agreement level. Any disagreement in study selection between the two authors was resolved by an independent third author (GM). Collation, management of potentially eligible records, and bibliographic citations obtained from the literature search were conducted using Endnote™, version X9 (Clarivate Analytics, *Philadelphia, USA*).

### Data extraction and outcomes of interest

Data extraction was performed independently by the two reviewers (DA and PS) according to the standardized and predefined data format, recording the following outcomes: 1) effectiveness of the technique; and 2) ease of clinical applicability. Accordingly, the following data were extracted from the full text articles:

1. Demographic data (age, gender, ethnicity, sample size, and skeletal discrepancy).
2. Characteristics of the study (study design, asymmetry criteria, and comparison groups).
3. Features of MSP construction (3D technique, software used, MSP type, nomenclature used, construction technique, and reference points).
4. Features of the analysis (landmark digitization, asymmetry assessment, reliability assessment, and measurement type)*.*

### Quality analysis

The methodological quality of each paper for the risk of bias and applicability was assessed using customized assessment criteria based on the *Quality Assessment of Diagnostic Accuracy Studies (QUADAS-2)* [19] (Supplementary Appendix 1). Two reviewers (DA and PS) rated each study independently and assigned a score of low, high, or unclear based on the information presented in the study.

### Level of evidence

The strength of the evidence for the included articles was determined using the *Grading of Recommendations Assessment, Development, and Evaluation (GRADE)* scale [20]. The level of evidence was categorized as high, moderate, low, or very low. The ratings were downgraded for the studies exhibiting serious or very serious concerns pertaining to publication bias, inconsistency, imprecision, indirectness, or risk of bias.

## Results

### Study selection

**Fig. 1:**
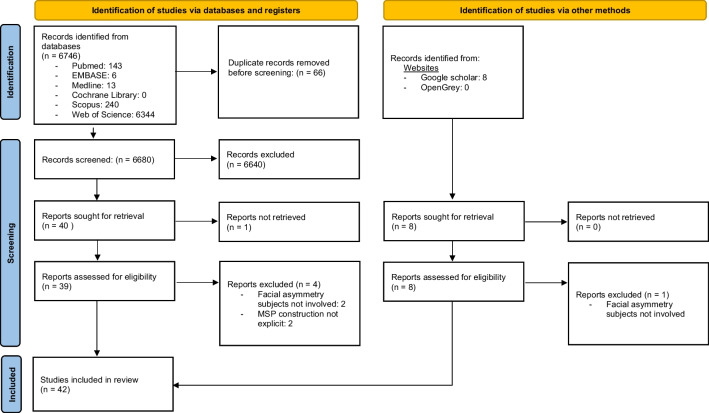
PRISMA flow diagram illustrating the study selection process

The PRISMA flowchart presented in Fig. 1 shows the study selection process. Initially, 6746 records were identified through a comprehensive search across six databases, and 8 records were identified from additional sources. After excluding 66 duplicates, the titles and abstracts of 6680 articles were screened. Of those, 6640 articles were excluded due to their irrelevance to the topic. Following initial screening, a total of 48 articles (40 from the database search and 8 from additional sources) were sought for full text retrieval; however, only 47 potentially eligible articles were assessed for full text review as the full text for one article was unavailable. Following a detailed review of the full text articles, an additional 5 studies were eliminated, and finally, 42 studies that met the inclusion criteria were considered suitable for qualitative analysis. A list of the excluded full text articles along with their justifications is presented in Supplementary Appendix 2. There was excellent inter-reviewer agreement for the study selection process, with Cohen’s κ values of 0.89.

### Study characteristics

The demographic and methodological characteristics of all the included studies are summarized in Tables 2 and 3. Between 2005 and 2023, a total of 42 studies were identified, focusing on estimating the mid-sagittal plane in patients with facial asymmetry. Out of the selected 42 studies, eight were computational studies [1, 3, 21–26]; six were observational [14, 15, 27–30]; 21 studies used retrospective analytical design [4, 9, 9, 12, 31–47]; three were experimental studies [17, 48, 49], and one study was ambispective in nature [50]. The age range of the patients in all the included studies was 7–57 years. Further, the included studies revealed diverse asymmetry criteria, such as a deviation of 1 mm to 4 mm in soft tissue chin position based on menton (Me), pogonion (Pog), or Gonion (Gn) [4, 12, 15, 24, 28, 30, 32, 9, 33–35, 38–45, 47–53].

**Table 2:** Demographic characteristics of the included studies

Author (year)	Study type	Sample size	Sex (M/F)	Mean age/age range (years)	Ethnicity	Skeletal discrepancy	Asymmetry criteria
Wong (2005)	Computational	1	F	17	Asian	Class III	n/r
Hartmann (2007)	Computational	n/r	n/r	n/r	Caucasian	n/r	n/r
AlHadidi (2011)	Observational	50	n/r	n/r	Caucasian	n/r	Chin > 2 mm Presence of cant of occlusal plane
Kim (2011)	Retrospective	102	F	21.551 ± 2.644/ 20–29	Asian	Class III	n/r
Baek (2012)	Retrospective	43 9, Class I 4, Class II 30, Class III	18/25	24.3 ± 4.4	Asian	Class I, Class II, Class III	n/r
Damstra (2012)	Observational	14 skulls 5, Asymmetry 9, Symmetry	n/r	n/r	Caucasian	n/r	Me > 4 mm
Berssenbrügge (2014)	Computational	50 faces	22 / 28	20–32	Caucasian	n/r	n/r
Wong (2014)	Retrospective	20	13 / 7	24.3	Asian	Condylar hyperplasia/hypoplasia; Hemifacial microsomia; Condylar tumour; Trauma related	Me > 4 mm/ 4°
Gateno (2015)	Experimental	1	M	n/r	Caucasian	n/r	n/r
Kim (2015)	Retrospective	24	12 / 12	22.5/ 18.2–29.7	Asian	n/r	Normal, 0 mm ≤ Me < 2 mm Mild, 2 mm ≤ Me < 4 mm Moderate, 4 mm ≤ Me < 8 mm Severe, 8 mm ≤ Me
Ryu (2015)	Observational	85			Asian	Class III	Me < 2 mm Me > 3 mm
		30, Control	15/15	24.30 ± 4.14			
		55, Asymmetry:					
		Hyperdivergent Hypodivergent	13/15 15/12	26.34 ± 3.14 28.96 ± 5.27			
Lee (2016)	Retrospective	35			Asian	n/r	Me < 2 mm Me ≥ 4 mm
		15, Symmetry	7/8	22.3 ± 3.3			
		20, Asymmetry	12/8	21.9 ± 3.4			
Shin (2016)	Retrospective	69	36 / 33	23.0 ± 4.1	Asian		Me > 3.6 ± 2.3 mm
		10, Class I	4 / 6	M, 22.9 ± 4.1 F, 23.0 ± 4.2		Class I,	
		12, Class II	5 / 7			Class II	
		47, Class III	24 / 23			Class III	
Song (2016)	Retrospective	29			Asian	Class III	Me > 4 mm
		16, CS	10/6	21.8 ± 2.2			
		13, POGS	5/8	21.2 ± 4.3			
Sangln An (2017)	Observational	30	n/r	25.7 ± 6.03/19–43	Asian	n/r	n/r
Dobai (2018)	Retrospective	60			Caucasian	n/r	n/r
		30, Group I	11 / 19	18–30			
		30, Group II	12/ 18	20–28			
Economou (2018)	Observational	21	7/14	13.5	Caucasian	Juvenile Idiopathic Arthritis	n/r
Jajoo (2018)	Computational	20 CT skull models	n/r	n/r	Caucasian	Horizontal/ Vertical condylar hyperplasia; Type 1/ Type 2 Hemifacial microsomia	n/r
Oh (2018)	Retrospective	60			Asian		Me > 2º
		30, Asymmetry	15/15	23.2 ± 3.8		Class I, Class III	
		30, Symmetry	13/17	24.6 ± 3.2		Class I, Class III	
Thiesen (2018)	Retrospective	120	41/79	30.58 ± 9.46/ 19–57	Caucasian	Class I	
		40, Relative asymmetry	10/30	31.10 ± 9.89/ 19–51			Gn < 2 mm
		40, Moderate asymmetry	15/25	30.57 ± 9.32/ 19–51			Gn = 2–4 mm
		40, Severe asymmetry	16/24	30.05 ± 9.35/19–57			Gn > 4 mm
Udomlarptham (2018)	Retrospective	37	15/22	25.76 ± 7.14	Asian	Class III	Me > 4 mm
Wong (2018)	Retrospective	59	n/r	n/r	Asian	n/r	Me > 4 mm / 4°
Zhang (2018)	Retrospective	12 6, Asymmetry 6, Symmetry	n/r	n/r	Asian	n/r	Me and Pog > 4 mm
Zheng (2018)	Experimental	30	14 / 16	18–34	Asian	n/r	Soft tissue chin > 1 mm
Choi (2019)	Retrospective	40 4, Class I 3, Class II 33, Class III	18/22	25.5, 19–42	Asian	Class I, Class II, Class III	n/r
Kwon (2019)	Retrospective	46	27/19	22 ± 4.8	Asian	Class III	Me > 4 mm
Tan (2019)	Computational	10	n/r	20–75	Asian	n/r	n/r
Vernucci (2019)	Retrospective	15 7, Symmetry 8, Asymmetry	6/9	16–52	Caucasian	Class I, Class III, Condylar hyperplasia, Hemifacial microsomia	n/r
Han (2020)	Experimental	29	15 / 14	23.1 ± 6.9	Asian	Class III	Me > 4 mm
Lee (2020)	Observational	43	21 / 22	23.0 ± 8.20	Asian		
		10, Group 1	4 / 6	24.1 ± 10.14		Class I	Me < 2 mm
		11, Group 2	5 / 6	22.3 ± 7.50		Class III	Me < 2 mm
		9, Group 3	5/4	23.2 ± 5.19		Class III	2 mm < Me < 4 mm
		13, Group 4	7 / 6	22.6 ± 9.58		Class III	Me ≥ 4 mm
Ortún-Terrazas (2020)	Computational	20	9 / 11	M, 7.9 F, 8.2	Caucasian	Unilateral crossbite	Minor < 0.3 mm; Moderate: malformations either in maxilla / mandible; Marked: maxillary and mandibular deformities + pronounced effect in the superficial soft tissue
Zhu (2020)	Computational	15	n/r	n/r	Asian	n/r	Me > 3 mm
Jo (2021)	Retrospective	38			Asian	n/r	Me > 4 mm
		23, PBO	9/14	22.57 ± 4.97/ 17–37			
		15, Grinding	9/6	21.80 ± 4.89/ 18–38			
Lv (2021)	Prospective	75		18–35	Asian	Class I, Class II	
		25, Class I symmetry	6/19	23.46 ± 3.99			Gn < 2 mm
		25, Class II symmetry	3/22	25.57 ± 4.55			
		25. Class II asymmetry	5/20	25.08 ± 3.59			Gn > 4 mm
Mangal (2021)	Retrospective	34	19/15	22.38 ± 5.20 /18–47	Asian	Class III	Me > 4 mm
Teng (2021)	Prospective	122	n/r	n/r	Asian	n/r	
		80, Asymmetry 42, Control					Chin > 2 mm
Teng (2021)	Prospective	40	n/r		Asian	High angle Class III	
		20, Experimental		22.10 ± 3.01/ 18–28			Me > 2 mm
		20, Control		24.10 ± 3.45/ 18–32			
Ajmera (2022)	Retrospective	42			Asian	Class III	
		21, Asymmetry	7/14	23.0 ± 3.4			Chin > 3 mm
		21, Control	7/14	23.0 ± 3.3			
Feng (2022)	Retrospective	60	n/r		Asian	n/r	
		30, Symmetry		26/ 20–32			Me < 2 mm
		30, Asymmetry		24.7/ 19–30			Me > 4 mm
Grissom (2022)	Ambispective	54	n/r	n/r	Caucasian	Goldenhar syndrome, Hemifacial microsomia, Mandibular hyperplasia, Mandibular hypoplasia, Unilateral condylar hyperplasia, Unilateral condylar destruction, Positional plagiocephaly, Juvenile arthritis	Chin > 4 mm
Hsiao (2022)	Computational	20	7/13	20–44	Asian	Craniofacial dysplasia	n/r
Ajmera (2023)	Retrospective	42			Asian	Class III	
		21, Asymmetry	7/14	23.0 ± 3.4			Chin > 3 mm
		21, Control	7/14	23.0 ± 3.3	n		

*Me* Menton, *Pog* Pogonion, *Gn* Gonion, *M* Male, *F* Female

**Table 3:** Methodological characteristics of the included studies

Author (year)	3D Technique	Software used	MSP Type	Nomenclature	MSP construction	Reference points	Asymmetry assessment	Landmark digitization	Comparison	Reliability assessment	Measurements type	Outcome	Remark / Conclusion
Wong (2005)	CT	n/r	Symmetry Plane	Best symmetry plane (BSP)	Mathematical optimization algorithm based	Landmark-independent	Symmetry value, representing the percentage of pixels that can be paired on both sides	Landmark-independent	BSP of midface vs BSP of mandible	n/r	Angular	75.6% ↓ in asymmetry value using BSP	Correction of BSP of the mandible might greatly alleviate the FA
												BSP of midface and mandible diverse by 3.125°	
Hartmann (2007)	FaceScan3D	Slim3D	Morphometric MSP	Symmetry plane	Iterative Closest Point (ICP) algorithm based OMR	Landmark-independent	Mean absolute distance between the original facial surface and mirror-image surface	Landmark-independent	3D facial surface data recordings performed consecutively vs those performed on different days	Mean deviation angle between the symmetry planes	Linear, Angular	Among three measurement sets: phi ^φ^, p < 0.05; d_abs_, p > 0.05	The method introduced here can be used to determine the symmetry plane and degree of asymmetry using 3D facial data with convincing reproducibility and without having to refer to landmarks
AlHadidi (2011)	CBCT	Insight SNAP	Cephalometric MSP		Plane passing through three median landmarks	Na-ANS-Ba	The 95th percentile surface distance measurement of each ROI	Manual	Mirroring using MSP vs mirroring using- registration based approach	Differences between repeated assessments of asymmetry	Linear	Mirroring using MSP = mirroring using registration, p > 0.05	Both mirroring techniques provided similar quantification of mandibular asymmetry
Kim (2011)	CT	InVivoDental	Cephalometric MSP	Cranial MSP	Plane crossing three landmarks	CG-apFO-apFS	Absolute value of ⟂ distance from MSP to the midpoint	Manual	Cranial MSP vs Facial MSP	ICC, r ≥ 0.978	Linear	Mean DCs 10–17 times > mean DFs, p < 0.001	The facial MSP was not in agreement with the cranial MSP Cranial MSP could exaggerate the result of the jaw deviation
				Facial MSP	Plane crossing CG and vertically bisecting a line formed by FZS on both sides	CG-FZS							
Baek (2012)	3D-CT	Simplant Pro	Cephalometric MSP	MSP	Plane passing through Na, Cl and Ba	Na-Cl-Ba	Distance of 3D landmarks to MSP	Manual	Four groups with distinct facial asymmetry features	ICC, 0.81 – 0.96	Linear	Group 1 ≠ Group 2 ≠ Group 3 ≠ Group 4, p < 0.05	Patients with asymmetry were classified into four statistically distinct groups according to their anatomic features
											Angular	Group 1 = 44%, caused by lateralization of mandibular body	
Damstra (2012)	CBCT	SimPlant®Ortho Pro 2.1	Cephalometric MSP		Plane passing through three midline structures	1: S–N-ANS 2: S–N-Me 3: LFM-ACP-N 4: S–N 5: Cg-ACP 6: ELSA-MDFM	Linear measurements (mean ± SD) compared between AG and SG	Manual + Digital	Morphometric MSP vs Cephalometric MSPs	Method error (mean = 0.39 mm; 95% CI = 0.31–0.47 mm)	Linear	1–3 and 6 vs Morphometric MSP, Absolute error (AE) > 1.00 mm	A morphometric approach to determine the MSP, might be more valuable for diagnosis and treatment planning of craniofacial asymmetry
					Plane passing through two midline structures + ⟂ to HRP								
										r = 0.845–0.999		4–5 vs Morphometric MSP, AE < 1.00 mm	
			Morphometric MSP		Procrustes analysis (PA) based OMR	SOF, MZF, FNM, FOM							
Berssenbrügge (2014)	3-camera fringe projection system	n/r	Morphometric MSP	Symmetry plane	ICP algorithm based OMR	Landmark-independent	3D asymmetry index (AI)	Landmark-independent	2D vs 3D methods	n/r	Linear	3D AI, 2.54 ± 0.718	An overall symmetry plane does not necessarily have to pass through facial midline points. This technique is assumed to give a better estimate of the facial symmetry plane than those that are based on only a few reference points or even solely on facial midline points
							2D AI					2D AI, 24.8 ± 7.02 2D AI vs. 3D AI, r = 0.294; p = 0.038	
							2D z-score					2D z-score vs. 3D AI, r = 0.567 p < 0.001	
							2D FA					2D FA vs. 3D AI, r = 0.104; p = 0.472	
Wong (2014)	CT	Self-developed imaging-processing software	Symmetry plane	Optimal Symmetry plane (OSP)	Mathematical optimization algorithm based	Landmark-independent	n/r	Landmark-independent	Traditional surgical plan compared with Matching OSP based surgical plan	n/r		Significant ↓ in mandibular deviation	The new method resulted in surgical plans that brought about significantly less postoperative mandibular deviation while maintaining a reasonable occlusion
											Linear	ADD, p = 0.046 PDD, p = 0.007	
											Angular	DA, p = 0.001	
Gateno (2015)	n/r	n/r	Morphometric MSP	Primal sagittal plane (PSP)	LAGER (Landmark Geometric Routine) algorithm based on PA	All facial landmarks	n/r	n/r	n/r	n/r	n/r	n/r	Primal sagittal plane should improve the correctness of our cephalometric measurements and surgical plans
Kim (2015)	CT	Vworks + Vsurgery	Cephalometric MSP	Midsagittal reference plane (MRP)	M1: Plane passing through two midfacial landmarks and ⟂to HRP and CRP	CG-P	Difference between x-coordinate (Δx), severity of asymmetry (SA), and direction of deviation (Dd) for four landmarks	Digital	M1 vs M2	n/r	Linear	Δx, M1 ≠ M2, p < 0.05 SA, M1 ≠ M2 Dd, M1 ≠ M2 for Na and L1 Location of landmark, M1 ≠ M2, p < 0.05	Location of midfacial landmarks, distance and direction of deviation, as well as the severity of asymmetry, may be influenced by the method of establishing the MRP
					M2: Plane passing through three midfacial landmarks	Op-CG-ANS							
Ryu (2015)	CBCT	OnDemand3D	Cephalometric MSP	MSP	Plane constructed with N and ⟂ to the line connecting bilateral frontozygomatic point	N-FZP	Angular and Linear distance of the landmarks from the MSP	Manual	Asymmetry vs Control group	ICC, 0.82 – 0.93	Linear	Shift, Yaw: Asymmetry ≠ Control, p < 0.05	Me deviation in skeletal Class III deformity with mandibular asymmetry is influenced by rotation of mandibular posterior dentofacial structures
											Angular	Shift, Yaw: Hyperdivergent > Control; Hypodivergent > Control, p < 0.01	
Lee (2016)	3D-CT	V-works	Cephalometric MSP	MRP	Plane passing through Op, Cg and ANS	Op-Cg-ANS	Distance of Me from the MRP	Manual	Location of the Me determined by PA cephalogram and 3DCT	n/r	Linear	Me deviation, PA cephalogram ≠ 3DCT Δx = 2.45 ± 2.03 mm, p < 0.05	In facial asymmetry analysis using 3D CT, the definition of facial asymmetry should be based on Me deviation on 3D CT, not on the cephalogram
Shin (2016)*	CBCT	Ondemand3D	Morphometric MSP	Symmetric MRP	PA based OMR	FO, FR, FS, FZM, GPC, HGC, Io,Or, Po, So, ZMS, A-point, ANS, Ba, INC,Na, NPC, Op, PNS, S, B-point, Me, G*	By comparing 1aandmark changes and differences in the amount of asymmetry between the original and symmetric configurations and 2 shapes produced by the 2 methods	Digital	Symmetric	ICC	Linear	Asymmetries measured by 3-landmark MRP > symmetric MRP	Statistical shape analysis confirmed that 3D-MRP constructed of Na, ANS, and PNS is compatible with the symmetric MRP and could be a valuable tool for evaluation of patients with FA
									MRP vs 3-landmark-based MRP	Intraexaminer, 0.924–0.941			
										Interexaminer, 0.811–0.916		SSED, SPD, 3-landmark MRP ≈ Symmetric MRP; p > 0.05	
			Cephalometric MSP	3-landmark-based MRP	Plane passing through three midline landmarks	Na-ANS-PNS	Total variations of asymmetry measurements according to 2 methods						
Song (2016)	CT	Invivo 5.4	Cephalometric MSP	MSP	Plane ⟂to FH plane and passing through N and S	N-S	Distance of Me from the MSP	Manual	CS vs POGS	ICC > 0.99	Linear	CS ≈ POGS, p > 0.05	POGS may be a clinically acceptable alternative to CS
Sangln An (2017)	3D CT	Simplant version 14.0	Cephalometric MSP		Plane passing through three landmarks while ⟂ to FH plane	1. FH-Na-Ba 2. FH-Na-S 3. FH-Cg-Ba 4. FH-Cg-S 5. Ba-Na-S 6. Ba-Cg-S 7. Ba-Na-ANS 8. Ba-Cg-ANS	Absolute values of differences in the measurement of Me deviation, ANS deviation, A-P line deviation	Manual	Eight different MSP configurations were compared	ICC, 0.91 – 0.95		MSP 1, showed smallest absolute values for	Using MSPs passing through 3 median landmarks in the cranial base can lead to underestimation of the asymmetry of Me, ANS, and the A-P line. The authors suggest using MSPs perpendicular to the FH plane or a plane passing through ANS in clinical practice
											Linear	AVDMe – 0.81 ± 1.33 AVDANS – 0.44 ± 0.66	
											Angular	AVDAP – 0.43 ± 0.59	
Dobai (2018)^#^	CBCT	CranioViewer software	Cephalometric MSP	Regression planes	Fifty planes were generated by a combination of unpaired landmarks, and paired cephalometric points	Combination of three unpaired landmarks and three paired cephalometric points	n/r	Digital	Regression planes vs Na-ANS-PNS (reference) plane	ICC > 0.9	Angular	Regression planes generated from unpaired landmarks and paired points had < 5° deviation from the reference plane	The N-ANS-PNS reference plane, which represents the ideal morphometric midplane, can be substituted by planes derived from the following landmark combinations: ANS-G-Ba, ANS-G-S, ANS-S-De, PNS-G-Ba, PNS-S-Ba, and PNS-ANS-G, and PNS-N-Ba
Economou (2018)	CBCT	Mimics	Cephalometric MSP	MRP	Plane ⟂ to the axial and coronal planes and passing through N	N	Distance of landmarks from the MRP	Manual	Hard tissue vs Soft tissue asymmetry	ICC, 0.74 – 0.98	Linear Angular	Pog showed largest deviation from MRP	Soft tissue pogonion and gonion were identified as the most appropriate landmarks to clinically predict hard tissue facial asymmetry. Facial asymmetries are most pronounced in the lower facial third in patients with juvenile idiopathic arthritis
Jajoo (2018)	3D CT	3D Studio Max	Morphometric MSP	PSP	LAGER algorithm based on PA	11 unpaired and 26 paired landmarks	n/r	Digital	Algorithm-generated MSP vs Ground truth	n/r	Linear Angular	For all the algorithm-generated MSPs, DistN < 1 mm, DisU1 < 1 mm, DistPg < 2 mm and θ < 2°	All the LAGER algorithm-generated MSPs qualified as clinically acceptable. LAGER algorithm can be used clinically to determine the MSP for patients with CMF deformities
Oh (2018)	CT	V-works	Cephalometric MSP	MRP	Plane ⟂to FH plane and passing through Cg and Op	Cg-Op	Linear and angular position of the condyle from MRP	Manual	Asymmetry vs symmetry groups	ICC, 0.98 – 0.99	Linear	Mediolateral condylar position, p > 0.05	In individuals with facial asymmetry, menton deviation is associated with the right/left differences caused by a smaller condyle on the deviated side
											Angular	Condylar angle, p > 0.05	
											Volumetric	p < 0.05	
Thiesen (2018)	CBCT	SimPlant Ortho Pro	Cephalometric MSP	MSP	Plane passing through N and Ba and ⟂to FH plane	N-Ba	Distance of landmarks from the MSP	Manual	Relative vs Moderate vs Severe asymmetry	ICC > 0.80	Linear Angular	Severe asymmetry: Contl side ≠ Dev side, p < 0.05	A great deviation of the mandibular dental midline may indicate severe skeletal asymmetry in Class I adults
Udomlarptham (2018)	CBCT	Simplant O & O	Cephalometric MSP	MSP	Plane ⟂to FH plane and passing through N and Ba	N-Ba	Distance of 3D landmarks to MSP	Manual	2DP vs 3DS	Measurement error:		2DP ≠ 3DS, p < 0.05	The deviated centre landmarks to the MSP improved significantly, and improved surgical outcomes were achieved through 3DS
										Linear, 0.43 – 0.92	Linear	Go to MSP, p < 0.05	
										Angular, 0.39º—0.85º	Angular	Yaw angle, p < 0.05	
Wong (2018)	CT	Self-developed imaging-processing software	Symmetry plane	OSP	Mathematical optimization algorithm based	Landmark-independent	Deviation angle and deviation distance formed by 2 OSPs in 3-dimensions	Landmark-independent	n/r	ICC, 0.99	Linear	ADD > PDD, p < 0.0001 ADD, 7.22 ± 4.12 mm	Plane-to-plane analysis system (closely matching the mandibular OSP to the midface OSP) will correct misalignment and generally achieve a satisfactory overall skeletal symmetry
											Angular	FDA > I, p = 0.03 Mean FDA, 3.80° ± 3.89° 83% patients had significant mandibular misalignment (deviation, ≥ 4° or 4 mm)	
Zhang (2018)	CBCT	Mimics	Morphometric MSP		Global registration	Preliminary MSP: N-S-Ba	Coordinate values of each landmark in 3D coordinate system	Digital	Interexaminer comparisons of the mean coordinate values of each landmark	ICC, > 0.9	Linear	No significant difference in coordinate values by both examiners	The MSPs constructed using the novel method were extremely stable and reliable. The accuracy of MSPs does not rely on the accuracy of other planes and the MSP are not influenced by maxillofacial deformities, orbital malformations, or even mild or moderate cranial asymmetry
						Final MSP: landmark-independent							
Zheng (2018)	CT	ProPlan CMF®	Cephalometric MSP	Orbital margin plane (OMP)	Plane passing through the midpoint of the NFS and ⟂ to FZ suture line	NFS	Distance of the landmarks to MSP	Digital	OMP vs SBP	Paired t-test, p = 0.873	Linear	Measurements in OMP < SBP, p < 0.05	OMP is more stable, accurate, and reliable, and therefore more suitable for the evaluation of FA
				Skull base plane (SBP)	Plane passing through three landmarks	S–N-Ba							
Choi (2019)	3D-CT	Mimics	Cephalometric MSP	MSP	Plane ⟂to AxP and passing through Cr and Cl	Cr-Cl	Distance of Me from the MSP	Manual	n/r	ICC, 0.91 – 0.99	Linear	Chin deviation correlated with mandibular length (r = -0.897) and mandibular body length (r = -0.318)	Treatment planning in patients with chin deviation should involve a careful evaluation of the asymmetry of the upper and middle facial thirds
Kwon (2019)	CBCT	Invivo 6	Cephalometric MSP	MSP	Plane ⟂to FH plane and passing through N and S	N-S	Similarity Index (SI) and Non-overlapping volume (NOV)	Digital	Mandibular and lower facial soft tissue measurements between Dev and, N-Dev sides at T1 and T2 using MSP and AMP	ICC > 0.99	Surface area, Volumetric	SI ↑ from 0.4 to 0.5, using MSP, and from 0.2 to 0.4, using AMP	SI and NOV can easily and intuitively evaluate overall 3D morphological discrepancies, especially 3D mandibular asymmetry
				Absolute mandibular midsagittal plane (AMP)	Plane passing through Me, B and G	Me-B-G*							
												NOV, using MSP ≈ AMP	
Tan (2019)	CT	Matlab	Symmetry plane	OSP	Oriented Bounding Box (OBB) → Mathematical translation + Mutual information method	Landmark-independent	n/r	Landmark-independent	Manual vs Semi-automatic	n/r	Linear	FAI, Manual ≈ Semi-automatic	Accuracy of semi-automatic method is almost equal to the accuracy of the doctor’s manual method
Vernucci (2019)	CBCT	Dolphin	Cephalometric MSP	Anatomical MSP	Plane passing through Na, PCM and Ba	Na-PCM-Ba	Distance of 3D landmarks to MSP	Manual	Anatomical MSP vs Median plane	n/r	Linear	Anatomical MSP accuracy > Median plane	Anatomical MSP can be used as a reliable reference plane for transverse measurements in 3D cephalometry in cases of symmetrical or asymmetrical malocclusion
				Median plane	Plane passing through the midpoint of inter-zygomatic distance	Zr-Zl	Inter-zygomatic distance on PA cephalograms					AMD ≈ 1 mm; Percentage difference < 3%	
Han (2020)*	CBCT	Invivo 6	Cephalometric MSP	Facial MSP	Plane passing through the landmarks while ⟂ to FH plane	N-S	SI using mirroring	Digital	Facial MSP vs modified MSP configurations	ICC > 0.99		SI using cmAMP > other MSPs, p < 0.05	The cmAMP plane best matches the two anterior segments of hemi-mandible symmetrically and is closest to Facial MSP after orthognathic surgery in skeletal Class III patients with FA
				AMP	Plane passing through Me, B and G	Me-B-G*						SI using cmAMP = SI using Facial MSP, p > 0.05	
				mAMP	Plane passing through the center point of Mf and ⟂ to the line connecting bilateral Mf	Mf					Linear		
				cmAMP	Plane was established at a point with highest SI and at the centre of bilateral Mf	Mf					Angular	The distance (1.15 ± 0.74 mm) and angle (2.02 ± 0.82◦), between Facial MSP and cmAMP < between Facial MSP and other MSPs, p < 0.05	
Lee (2020)	CBCT	Invivo 5.4	Cephalometric MSP		Plane passing through median landmarks and ⟂ to FH plane passing through bilateral landmarks	Reorientation method (RM)1: Cg-Ba and Ror-Rpo-Lor	⟂distances from each landmark to three different MSPs	Digital	Three MSPs established by different RMs were compared	ICC > 0.9	Linear	Mean absolute difference (MADs), RM 1 ≈ RM 2 ≈ RM 3, p < 0.05	Although the differences in distance among the three MSPs were minor, the MSP established by RM 1 best approximated the true symmetrical MSP. This MSP could be implemented as the reference plane for the diagnosis of FA regardless of the extent of chin deviation
						RM 2: N- IF-Ba and Ror -Rpo						MAD scores of RM 2 and RM 3 were 2–3 times > RM 1 (0.20 ± 0.10 mm)	
						RM 3: N, ANS, PNS and Ror -Rpo							
Ortún-Terrazas (2020)^†^	CBCT	i-CAT	Morphometric MSP	Sagittal midplane	Principal Component Analysis (PCA) + ICP algorithm based OMR	Me, PhT, and G†	Distance from the midplane of the mandible (ManDev) and the distance from the midplane of the Me (MeS), to the sagittal midplane respectively	Manual	n/r	n/r	Linear	Bilateral measurements of cross side ≠ non-cross side, p < 0.05	ManDev was more representative of the asymmetry than the MeS. PCA-based algorithm identified accurately and objectively the sagittal midplane in each subject, allowing the subsequent 3D-diagnosis workflow
												Significant malformations in mandibular ramus length (0.0086), maxillary palate width (0.0481), condylar head width (0.0408) in patients with severe asymmetry (jaw deviation > 0.8 mm)	
Zhu (2020)	Face Scan 3D	Geomagic Studio 2013	Morphometric MSP	PA Symmetry Reference Plane (SRP)	PA based OMR	Thirty-two anatomical landmarks	n/r	Manual	PA SRP vs WPA SRP vs Ground truth SRP	n/r	Linear	Global and regional position errors, WPA SRP < PA SRP	This novel automatic algorithm, based on weighted anatomic landmarks, can provide a more adaptable SRP than the standard PA algorithm when applied to severe mandibular deviation patients and can better simulate the diagnosis strategies of clinical experts
				WPA SRP	Weighted PA (WPA) based OMR								
				Ground truth SRP	Professional (regional ICP) algorithm based OMR						Angular	FAI error and Angle error, WPA SRP ≈ Ground truth SRP	
Jo (2021)	CBCT	OnDemand3D	Cephalometric MSP	MSP	Plane ⟂to FH plane and passing through N and S	N-S	Distance of landmarks from the MSP	Manual	PBO vs GR	ICC, 0.82 – 0.92	Linear	PBO ≠ GR, p < 0.014	PBO is recommended over the grinding method for patients with severe facial asymmetry
Lv (2021	CBCT	Dolphin 3D	Cephalometric MSP	MSP	Plane ⟂ to the horizontal plane and passing through N and Ba	N-Ba	Distance of landmarks from the MSP	Manual	Asymmetry vs Symmetry groups	ICC > 0.95	Linear	Dev ≠ Contl side Co-MSP, p = 0.030 Go-MSP, p = 0.003	Patients from the Class II asymmetry group showed significant differences between measurements on the contralateral and deviated sides,
											Angular	∠C-MSPº: p = 0.022	
Mangal (2021)	CBCT	Invivo 6	Cephalometric MSP	cmAMP	Plane ⟂to FH plane and passing through N and S	N-S	SI and NOV	Digital	SI and NOV between each segment and total mandible at T1 and T2	Paired t-test, p < 0.001	n/r	T2 – T1: SI score, between total mandible and anterior (r = 0.34, p = 0.044) and middle (r = 0.85, p < 0.001) segments	cmAMP based ToSS protocol allows accurate identification of the region of deformity of the mandible and minimizes residual asymmetries
Teng (2021)	CBCT	Mimics	Cephalometric MSP	MSP	Plane passing through S, N and Anterior Nasal Spine	S–N-ANS	Deviation between mental apex of the chin and midsagittal plane in the coronal position	Manual	Jaw deformity vs Control	n/r	Linear	Jaw deformity ≠ Control, p < 0.001	A positive correlation was found between the inclination of the occlusal plane and the degree of jaw deformity, with a linear relationship between them
											Angular	Positive correlation between MSP and Occlusal plane, Mental apex of chin Max. and Mand. Incisor midline ≠ MSP, p < 0.001	
Teng (2021)	CBCT	Mimics	Cephalometric MSP	MSP	Plane passing through S, N and Anterior Nasal Spine	S–N-ANS	Deviation between mental apex of the chin and midsagittal plane in the coronal position	Manual	Experimental vs Control	ICC, 0.97–0.99	Linear	Experimental ≠ Control, p < 0.05	Certain characteristics of mandibular symmetry and the occlusal plane were found in patients with high-angle skeletal class III malocclusion and jaw asymmetry
											Angular	Positive correlation between Mandibular deviation and Occlusal plane, r = 0.860, p < 0.001	
Ajmera (2022)	CBCT	3D Slicer	Cephalometric MSP	MSP	Plane ⟂ to HP and passing through N and S	N-S	Distance of landmarks from the MSP	Manual	Asymmetry vs Control	ICC, 0.90–0.99	Linear	T2, Significant correction of Me deviation, p < 0.001	Despite significant correction after bimaxillary surgery, asymmetry persisted at several sites, thereby requiring secondary correction
												Residual asymmetry at MF, p < 0.001	
Feng (2022)	CBCT	Mimics	Morphometric MSP	MSP_ACB_	Global registration		Euclidean distance of midline points to the MSP	Digital	MSP_ACB_ compared with MSP_morph_	ICC = 0.99	Linear	MSP_ACB_ ≈ MSP_morph,_ p > 0.05	MSP_ACB_ is reliable for patients with or without facial asymmetry in maxillofacial asymmetry analysis
				MSP_morph_	PA	SOF, MZF, FNM, FOM						Stability, MSP_ACB_ > MSP_morph,_ p < 0.05	
Grissom (2022)	CT/CBCT	Anatomic Aligner	Cephalometric MSP	MSP	Plane ⟂to axial plane and passing through Na and Ba	Na-Ba	n/r	Manual	Axial-plane-first vs midsagittal-plane-first	ICC > 0.89	n/r	Facial reference frames defined by the midsagittal plane-first method ≠ axial-plane-first method, p = 0.001	Midsagittal plane-first sequence improves the facial reference frames compared with the traditional axial-plane-first approach
			Morphometric MSP	PSP	Iterative WPA	Landmark-independent							
Hsiao (2022)	CT	n/r	Cephalometric MSP	LSP	Plane passing through CG, ANS and mid point of OrR_OrL	CG-ANS-mid OrR-OrL	Hausdorff distance (HD), Jaccard similarity coefficient (JSC) and Dice similarity coefficient (DSC)	Manual	Landmark-based vs Surface-based vs Voxel-based	n/r	Linear	HD: OSP < LSP < SSP JSC and DSC:OSP > LSP = SSP	The voxel-based method proposed in this research is a robust and reliable approach to evaluate the symmetry plane for severe asymmetry cases
			Morphometric MSP	SSP	PCA + ICP algorithm	Landmark-independent		Landmark-independent					
				OSP	Voxel-based symmetry plane	Landmark-independent		Landmark-independent					
Ajmera (2023)	CBCT	3D Slicer	Cephalometric MSP	MSP	Plane ⟂ to HP and passing through N and S	N-S	Asymmetry index (AI)	Manual	Asymmetry index vs Asymmetry scores	ICC, 0.90–0.99	Linear	MPA≈CDM > PA	Modified Procrustes analysis is proficient in evaluating cranio-facial asymmetry with more valid clinical representation and has potential applications in assessing asymmetry in a wide spectrum of patients
		MATLAB	Morphometric MSP		Clinically derived midline	N-S	Asymmetry scores	Digital					
					PA	All landmarks							
					Modified PA	Por-Or							

*CT* Computed Tomography, *CBCT* Cone Beam Computed Tomography, *MSP* Mid Sagittal Plane, *HRP* Horizontal reference plane, *FH* Frankfurt Horizontal, *mAMP* Modified absolute mandibular midsagittal plane, *cmAMP* Computed modified absolute mandibular midsagittal plane, *OMR* Original and Mirrored image Registration, *CRP* Coronal reference plane, *Na/N* Nasion, *ANS* Anterior Nasal Spine, *Ba* Basion, *CG/Cg/Cr* Crista galli, *apFO* averaged point of bilateral Foramina Ovale (FO), *apFS* averaged point of bilateral Foramina Spinosum (FS), *FZS/Zr/Zl* Frontozygomatic suture (left and right), *Cl/ACP* Midpoint of anterior clinoid process, *S* Sella, *Me* Menton, *LFM* Lateral foramen magnum, *ELSA* Foramen spinosum midpoint, *MDFM* Middorsal point of the anterior margin of the foramen magnum, *SOF* Supraorbital foramen, *MZF* Medial zygomaticofrontal suture (ZFS), *FNM* Frontonasomaxillare, *FOM* Fontorbitomaxillare, *P* Prechiasmatic groove, *Op* Opisthion, *FZP* Frontozygomatic point, *FR* Foramen Rotundum, *GPC* Greater palatine canal, *HGC* Hypoglossal canal, *Io* Infraorbitale, *Or* Orbitale (Right/Left), *Po/Por* Porion (Right /Left), *So* Supraorbitale, *ZMS* Zygomaticomaxillary suture, *INC* Inferior nasopalatine canal, *NPC* Nasopalatine canal, *PNS* Posterior Nasal Spine, G* Genial tubercle, *B-point* Deepest point between the chin and the mandibular incisors, *NFS* Nasofrontal suture, *PCM* Midpoint between the posterior clinoid processes of the sella turcica, *Mf* Mental foramen, *IF* Incisive foramen, *PhT* Pharyngeal tubercle, *G†* Glabella, *ROI* Region of Interest, *AG* Asymmetry group, *SG* Symmetry group, *2D AI* Two-dimensional Asymmetry Index, *2D z-score* Two-dimensional z-score, *2D FA* Two-dimensional Facial Asymmetry, *CS* Conventional orthognathic surgery, *POGS* Preorthodontic orthognathic surgery, *2DP* 2D Planning menthod, *3DS* 3D surgical simulation, *PBO* Posterior bending osteotomy, *GR* Grinding group, *ICC* Intra-class correlation coefficient, *Phi *^*φ*^ Angular deviations, *dabs* Degree of; asymmetry, *DC* Distance from the midpoint to the cranial MSP, *DF* Distance from the midpoint to the facial MSP, *3D AI* Three-dimensional Asymmetry Index, *ADD* Anterior deviation distance, *PDD* Posterior deviation distance, *DA* Deviation Angle; difference; Δx Difference of the measurements, *L1* Point between left and right mandibular incisors, *SSED* Sum of the squared Euclidean distances, *SPD* Squared Procrustes distance, *AVDMe* Absolute value of differences in menton deviation, *AVDANS* Absolute value of differences in ANS deviation, *AVDAP* Absolute value of differences in A-P line deviation, *Go* Gonion, *DistN* Distance between N and MSP, *DistPg* Distance between Pg (Pogonion) and MSP, *ADD* Anterior deviation distance, *PDD* Posterior deviation distance, *FDA* Frontal deviation angle, *HDA* Horizontal deviation angle, *FAI* Facial Asymmetry Index FA, *AMD* Absolute Mean Difference, *∠C* Condyle angle, *T2* Post-surgery

### Study quality assessment

**Fig. 2:**
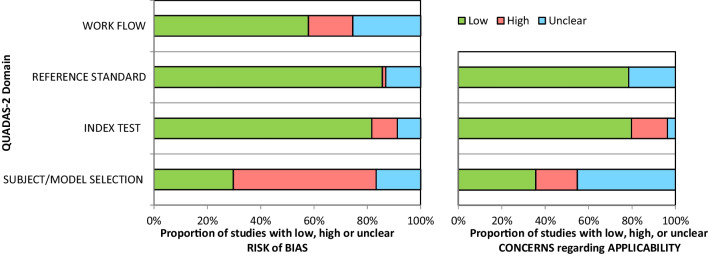
Methodological quality and risk of bias assessment of the included studies

The results for the methodological quality and risk of bias assessment have been presented in Fig. 2. All the included studies were representative of the target population; nevertheless, owing to the retrospective, observational, experimental, or computational nature of the included studies, concerns regarding the risk of bias were rated to be relatively ‘high’ in the subject/model selection domain. Within the index test domain, seven studies failed to explicitly define facial asymmetry [3, 16, 17, 22, 31, 9, 46] and four studies inadequately described the process for MSP construction [16, 21, 9, 33]. As a result, these studies were considered to have a ‘high’ risk of bias. However, the overall risk of bias in the index test domain was assessed as ‘low’. Concerning the risk of bias in the reference standard domain, most of the studies were considered to have a ‘low’ risk. With regard to the risk of bias in the domain of workflow, the validity and reliability of MSP construction were questionable in 13 of the included studies [9, 16, 17, 21–26, 32, 41, 46, 52], and therefore considered to have an ‘unclear/high’ risk of concern. In addition, 19 of the included studies [4, 15–17, 22–25, 28, 29, 31, 9, 33, 39, 43, 44, 49, 50] either did not report the specific skeletal discrepancy or focused solely on a particular type, such as Class III malocclusion, juvenile idiopathic arthritis, or craniofacial dysplasia. As a result, the MSP construction methodology described in those 19 studies may not be applicable to all types of facial asymmetry conditions and therefore, they were rated as having a ‘high’ risk of bias. In general, concerns regarding the risk of bias in the domain of workflow were relatively ‘low’.

Concerns about applicability were deemed to be ‘unclear’ in the subject/model selection domain since 19 of the included studies [4, 14, 15, 22, 23, 9, 24, 25, 28, 32–34, 36–39, 41, 46, 49, 52] lacked a detailed description of the sampling unit, including the type of skeletal discrepancy, asymmetry criteria, or the patient’s age and gender. The reproducibility of the constructed MSP was found to be questionable in 13 of the included studies [16, 17, 21–26, 32, 9, 41, 46, 52] as they failed to report the reliability assessment, which led to a rating of ‘high’ risk of concern; however, the overall risk was judged to be ‘low’ in the index test domain. Regarding the reference standard domain, nine studies with ambiguous reference standards were considered to have ‘unclear’ risk [17, 26, 31, 38, 39, 41, 49, 50, 52], while the majority of the studies were deemed to have ‘low’ risk. The Supplementary Figure includes a detailed presentation of the study quality and risk of bias assessment.

Table 4 provides the evidence profile for the outcomes examined in this study. The evidence quality was ‘low’ for the effectiveness of the technique and ‘moderate’ for the ease of clinical applicability.

**Table 4:** Evidence profile for the outcomes studied

Certainty assessment							Impact	Certainty	Importance
№ of studies	Study design	Risk of bias	Inconsistency	Indirectness	Imprecision	Other considerations			
Effectiveness of the technique (assessed with: Reliability assessment, Study outcomes, etc.)									
42	observational studies	not serious	serious^a^	not serious	serious^b, c^	strong association all plausible residual confounding would reduce the demonstrated effect	Cephalometric MSP and landmark dependent morphometric MSP are equally effective methods for MSP construction	⨁⨁◯◯ Low	CRITICAL
Ease of clinical applicability (assessed with: Technique simplicity, automation, etc.)									
42	observational studies	not serious	serious^d^	not serious	not serious	very strong association	A fully automated MSP construction method may be more practical for clinical application	⨁⨁⨁◯ Moderate	CRITICAL

*CI* confidence interval

Explanations

^a^.Methodological heterogeneity may exist due to differences in skeletal discrepancy, 3D imaging techniques, software used, MSP construction methods, or due to technique validity

^b^.13 studies had questionable internal validity as they failed to report the reliability assessment

^c^.Compatibility, user-friendliness, and technical simplicity of the software were questionable in 6 studies as they failed to report the software utilized

^d^.Methodological heterogeneity may exist due to differences in 3D imaging techniques, software used, technique automaticity, or landmark digitization

### Effectiveness of the technique

The majority of the included studies [12, 14, 27–31, 9, 32, 36, 37, 39–49, 51–53] constructed a cephalometric MSP, while eight studies computed a morphometric MSP [3, 17, 21–24, 34, 38] for the assessment of asymmetry (Table 3). In addition, five studies employed both cephalometric and morphometric MSP construction methods [4, 15, 25, 35, 50], while four studies assessed asymmetry using a symmetry plane [1, 26, 9, 33]. For the cephalometric MSP construction, N-S was the most consistently used landmark combination [12, 28, 35, 39, 40, 43, 47, 48]. Whilst morphometric MSP was determined using modified Procrustes Analysis [35], and Global Registration [34, 38]. The use of the morphometric MSP was found to be accurate and suitable for the evaluation of facial asymmetry in six studies [3, 4, 15, 34, 35, 38]. In particular, based morphometric MSPs showed extremely stable and reliable results (*ICC* > *0.9)* for the estimation of facial asymmetry.

### Ease of clinical applicability

A semi-automatic technique that involves digital landmarking was employed for cephalometric and morphometric MSP construction in nine studies [23, 24, 28, 31, 32, 40, 47–49], whereas a fully automated and landmark-independent technique for morphometric MSP and symmetry plane construction was reported in seven studies [3, 16, 21, 25, 9, 33, 50]. The fully automated MSP construction method was reported to accurately measure the degree of asymmetry and achieve satisfactory skeletal symmetry, as asserted by the authors in their respective studies (Table 3).

## Discussion

Symmetry is a fundamental aspect of a face’s ability to be regarded as socially alluring [54] and in order to achieve optimal function and facial esthetics, orthognathic surgery is performed to correct the underlying maxillomandibular deformity through osteotomies and repositioning of the maxilla-mandibular complex [10, 55, 56]. Management of a three-dimensional compound problem like facial asymmetry requires a realistic and composite approach, which entails modifying the roll, yaw, and pitch of the maxillo-facial structures [28]. Since patients are critical to any changes in their facial appearance following orthognathic surgery, a precise surgical outcome prediction is crucial. Considering the heterogeneity in the aetiology, pathology, and site of facial asymmetry, clinicians are compelled to follow a multifactorial, stepwise decision-making process. The first stage in this process is the establishment of an accurate MSP. Previous studies have substantiated that if MSP does not correspond to the external facial structure (facial morphology) and internal structures (skeletal structures), it provides fallacious information, thus affecting treatment planning [15, 49]. Therefore, the construction of an accurate MSP is essential not only for quantifying the degree of symmetry between the right and left sides of the face but also for arriving at the correct diagnosis.

A comprehensive systemic review of the literature revealed that the construction of the MSP is most commonly based on three techniques: cephalometric MSP, morphometric MSP, and a symmetry plane. Cephalometric MSP is a technique for constructing a mid-sagittal plane (MSP) that relies on anatomical landmarks and involves digitizing these landmarks manually or digitally. The morphometric MSP technique can be undertaken semi-automatically using landmarks or fully automatically without relying on landmarks. The symmetry plane, on the other hand, is a landmark-independent technique for constructing an MSP that can be done semi-automatically or automatically. In the semi-automatic approach, clinicians manually select a seed point in the CT image for segmentation using the "region growing" method. This is followed by extracting the "initial symmetry plane", which is then used with algorithms to calculate the "optimal symmetry plane" [26]. In contrast, the fully automatic technique of the symmetry plane relies solely on algorithms. It involves voxel pairing and mathematical optimization to determine the "optimal symmetry plane", eliminating the need for landmark identification [33]. Where landmark-dependent approaches utilized various paired or unpaired landmark combinations such as Nasion (N), Sella (S), Anterior Nasal Spine (ANS), Basion, and Crista Galli for the MSP construction, landmark-independent methods employed different mathematical algorithms, including the Iterative Closest Point (ICP) algorithm, the LAGER (Landmark Geometric Routine) algorithm, the Oriented Bounding Box (OBB) algorithm, Procrustes Analysis (PA), modified Procrustes Analysis, Weighted Procrustes Analysis (WPA), Principal Component Analysis (PCA), Global Registration method, Clinically derived midline, and Voxel-based method. Despite the fact that a myriad of nomenclatures have been used to represent various MSP construction methods (Table 3), a detailed analysis of the included studies revealed that all the MSPs fell into one of the three MSP construction approaches. Representing MSPs with multiple names in different studies can be misleading not only to the readers but also to the clinicians. Therefore, systematic reporting of various MSP types and nomenclatures becomes indispensable.

### Effectiveness of the technique

The cephalometric method has been evidenced to be reliable, highly familiar, and a simple technique, as head orientation has no influence on the landmark placement and is therefore convenient in the clinical setting [4, 28]. The present review demonstrated that cephalometric MSP based on N-ANS-PNS was consistent with the symmetric mid-sagittal reference plane [4]. However, a plane passing through the stable landmarks N-S [12] and perpendicular to the horizontal reference plane would be a more appropriate approach if damage or deviation of the ANS were suspected. Ajmera et.al, in their study, utilized N-S based cephalometric MSP and reported it to be equally effective as a clinically derived midline for facial asymmetry estimation [35]. Despite its several advantages, the cephalometric method may seem challenging due to the inherent limitations associated with its landmark digitization step. For instance, midline structures may tip from the true plane of symmetry; stable landmarks need to be identified that are unaffected by asymmetry; errors associated with manual or digital landmark annotation; and the reproducibility of the identified landmarks [4, 15]. The morphometric technique, on the other hand, was developed to overcome some of the shortcomings of the cephalometric method [15]. The strength of the morphometric method is its capacity to use the external face characteristics, which serve as a framework of perceptiveness for asymmetry, to ascertain the true plane of symmetry [15, 57]. Damstra et al. compared cephalometric and morphometric MSPs and suggested using a morphometric approach based on intact regions of the skull, unaffected by asymmetry, to determine MSP [15]. Likewise, Hsiao et al. reported voxel-based morphometric MSP as a more reliable approach compared to the cephalometric method, due to the latter’s dependence on the operator’s skill in identifying landmarks, whereas the former is less prone to deviation even in cases of severe asymmetry [25]. In contrast, a recent study by Ajmera et al. found cephalometric and morphometric approaches (clinically derived midline and modified Procrustes analysis) to be equally effective [35]. The authors further reported that the modified Procrustes method is a practical alternative to conventional Procrustes analysis for evaluating asymmetry. Conventional Procrustes analysis considers all landmarks to achieve an optimal fit, whereas the modified Procrustes method only utilizes four stable landmarks in the upper facial region (bilateral porions and orbitales) that are minimally influenced by asymmetry to achieve the best fit. This was in agreement with Shin et al.’s findings, who reported analogous results for asymmetry measurements (*p* > 0.05) using morphometric and cephalometric MSP construction approaches [4]. Zhu et al. in their study, compared three different algorithms for morphometric MSP construction and reported that weighted PA-based MSP was a promising approach for cases of severe mandibular deviation [24]. Assessing the various morphometric approaches previously employed, Global registration [34, 38] and modified PA [35] methods were found to be more robust approaches for asymmetry estimation. This is because the Global registration method utilizes a stable anterior cranial base for registration, preventing the influence of other asymmetric regions of the skull, whereas modified PA utilizes four stable landmarks (bilateral porion and orbitale) that are least affected by asymmetry to achieve the ‘best fit’. Interestingly, PA, which has been used in numerous prior investigations, was found to exhibit a masking effect that reduces asymmetry characteristics and may not accurately reflect the true clinical situation [35]. 

The reliability of the approach is critical for the clinical effectiveness of the MSP construction technique. In the present review, the reliability of the technique was critically analyzed, which revealed that several studies [1, 9, 17, 21–24, 32] failed to report the reliability assessment despite concluding with encouraging results. For instance, Wong et al. [16, 9, 33] advocated a novel automated and landmark-independent method of MSP construction referred to as the “*Symmetry Plane*”. Nevertheless, the effectiveness of their technique can be deemed questionable because the information regarding the software used was only briefly described and a reliability assessment was not performed.

### Ease of applicability

For routine clinical use, MSP construction needs to be simple, user-friendly, and ideally fully automatic. Current landmark-dependent methods are simple and user-friendly, but at the same time, they are also technique-sensitive, as they rely on manual or automatic landmarking. While manual digitization is subjective and time-consuming, automatic landmarking may not locate the landmarks precisely and may introduce further errors during asymmetry evaluation. When employing a morphometric approach, the cost of the software and hardware and the need for additional training should not be overlooked [4, 15, 58]. Although the morphometric approach may be quicker to implement, it may not be cost-effective. On the other hand, as the technique is less landmark-dependent and more reliable, it may improve diagnosis and reduce treatment planning time. In this regard, voxel-based morphometric MSP [25] could be a viable option for asymmetry assessment. Another fully automated approach, based on a ‘Symmetry plane’ [9, 16, 33] although showed promising results for routine clinical use, may not be applicable in a routine clinical context owing to the inaccessibility of the in-house developed software.

### Limitations and future outlook

Despite a comprehensive search and selection of specific studies, this review was limited due to the methodological heterogeneity observed across the included studies, which precluded performing a meta-analysis. Additionally, a lack of standardized assessment of facial asymmetry was noted, which may have had an impact on the findings. In addition, many of the included studies did not adequately demonstrate the accuracy of the constructed MSP. Future prospective studies with an emphasis on the impact, accuracy, and clinical applicability of MSP construction techniques in cases of facial asymmetry are required.

## Conclusion

Achieving favorable treatment outcomes and patient satisfaction in cases of facial asymmetry relies on accurate pre-operative planning. This systematic review highlights the importance of precisely determining the MSP during diagnosis and treatment planning. Despite significant methodological variations across the included studies, the following conclusions can be drawn:

1. Provided that stable landmarks are used, both cephalometric and morphometric methods for MSP construction are equally effective and offer the closest approximation to the true symmetrical MSP.
2. Among the various cephalometric and morphometric methods, a cephalometric MSP constructed using stable landmarks such as N-S and perpendicular to the FH plane provides optimal estimation of facial asymmetry. In terms of morphometric approaches, both global registration and modified PA methods are robust approaches for estimating asymmetry.
3. From a clinical applicability perspective, a fully automated voxel-based morphometric MSP holds promise as a viable option for routine clinical use.

The findings presented in this review will assist clinicians in selecting the most suitable method for MSP construction, leading to improved treatment planning and ultimately more favorable treatment outcomes.

### Supplementary information

Below is the link to the electronic supplementary material.

Supplementary file1 (DOCX 19 KB)

Supplementary file2 (DOCX 21 KB)

Supplementary file3 (PDF 109 KB)

## Data Availability

The datasets used and/or analysed during the current study are available from the corresponding author on reasonable request.
