# Supplementary material for: Establishment of the mid-sagittal reference plane for three-dimensional assessment of facial asymmetry: a systematic review: Establishment of the mid-sagittal reference plane: a systematic review
Source: Clin Oral Investig. 2024 Apr 5;28(4):242. doi: 10.1007/s00784-024-05620-7 (PMC10995046; doi:10.1007/s00784-024-05620-7)
Supplement: Supplementary file 1 — Supplementary file1 (DOCX 19 KB) [file 784_2024_5620_MOESM1_ESM.docx]

**Supplementary Appendix 1.** Description of rating guidelines across various QUADAS-2 domains for the assessment of methodological quality of the included studies

| **Domain** | **Subject/ model selection** | **Index test** | **Reference standard** | **Work-flow** |
| --- | --- | --- | --- | --- |
| ﻿*Concerns about Risk of bias (high, low, or unclear)* | If likely representative of the target population | If clinical findings, or any other assessment clearly defined FA | Selected reference (expert’s judgment/ clinical/ automated/ analytical measures ) likely to provide correct diagnosis and quantification of FA | ﻿Did all patients/models receive the same reference standard? |
|  | If study design:  1. Prospective  2. Retrospective/ Computational/ Observational/ Experimental | If MSP construction,  acquisition, described in sufficient detail | Reference standard results interpreted without knowledge of the results of the constructed MSP | ﻿If the MSP construction was valid and reliable |
|  |  | If study outcomes are indicative of distinct estimation of FA using the constructed MSP |  | ﻿Can MSP construction approach be applied indiscriminately to all the FA subjects |
| ﻿*Concerns about Applicability (high, low, or unclear)* | ﻿Subject/sampling unit good representative of facial asymmetry and  described in detail | Diagnostic index tests relevant to the research question (MSP) | Selection of reliable reference standard: expert’s judgment/ clinical/ automated/ analytical measures, etc. |  |
|  |  | If constructed MSP was reproducible |  |  |
