# Supplementary material for: Establishment of the mid-sagittal reference plane for three-dimensional assessment of facial asymmetry: a systematic review: Establishment of the mid-sagittal reference plane: a systematic review
Source: Clin Oral Investig. 2024 Apr 5;28(4):242. doi: 10.1007/s00784-024-05620-7 (PMC10995046; doi:10.1007/s00784-024-05620-7)
Supplement: Supplementary file 2 — Supplementary file2 (DOCX 21 KB) [file 784_2024_5620_MOESM2_ESM.docx]

**Supplementary Appendix 2.** ﻿List of excluded studies

|  | **Excluded studies** | **Reasons for exclusion** |
| --- | --- | --- |
| 1. | De Momi et al. (2006) | Facial asymmetry subjects not involved[1] |
| 2. | Shetye et al. (2021) | MSP construction not explicit[2] |
| 3. | Coban et al. (2021) | Facial asymmetry subjects not involved[3] |
| 4. | Arpaci et al. (2022) | MSP construction not explicit[4] |
| 5. | Yoshikawa et al. (2022) | Facial asymmetry subjects not involved[5] |
| 6. | Zhu et al. (2022) | Full text not available[6] |

**References:**

De Momi E, Chapuis J, Pappas I, Ferrigno G, Hallermann W, Schramm A and Caversaccio M (2006) Automatic extraction of the mid-facial plane for cranio-maxillofacial surgery planning. Int J Oral Maxillofac Surg 35:636-42. doi: 10.1016/j.ijom.2006.01.028

Shetye PR, Grayson BH and McCarthy JG (2021) Correction of Severe Facial Asymmetry in Patients With Unilateral Craniofacial Microsomia Using Computer-Aided Design/Computer-Aided Manufacturing Technology: An Evaluation of Postsurgical Results. Journal of Craniofacial Surgery 32:2416-2420. doi: 10.1097/scs.0000000000007949

Çoban G, Yavuz İ and Demirbaş AE (2021) Three-dimensional changes in the location of soft tissue landmarks following bimaxillary orthognathic surgery. Journal of Orofacial Orthopedics 82:257-265. doi: 10.1007/s00056-021-00279-1

Arpaci MF, Ozbag D, Aydin S, Senol D, Baykara RA and Cicek IB (2022) Evaluation of the relationship between nasal septal deviation and development of facial asymmetry with anthropometric measurements depending on age. International journal of pediatric otorhinolaryngology 159. doi: 10.1016/j.ijporl.2022.111207

Yoshikawa H, Tanikawa C, Ito S, Tsukiboshi Y, Ishii H, Kanomi R and Yamashiro T (2022) A three-dimensional cephalometric analysis of Japanese adults and its usefulness in orthognathic surgery: A retrospective study. Journal of Cranio-Maxillofacial Surgery 50:353-363. doi: 10.1016/j.jcms.2022.02.002

Zhu Y, Fu X, Zhang L, Zheng S, Wen A, Xiao N, Wang Y and Zhao Y (2022) A mathematical algorithm of the facial symmetry plane: Application to mandibular deformity 3D facial data. Journal of Anatomy 240:556-566. doi: 10.1111/joa.13564
