# Supplementary material for: Establishment of the mid-sagittal reference plane for three-dimensional assessment of facial asymmetry: a systematic review: Establishment of the mid-sagittal reference plane: a systematic review
Source: Clin Oral Investig. 2024 Apr 5;28(4):242. doi: 10.1007/s00784-024-05620-7 (PMC10995046; doi:10.1007/s00784-024-05620-7)
Supplement: Supplementary file 3 — Supplementary file3 (PDF 109 KB) [file 784_2024_5620_MOESM3_ESM.pdf]

Supplementary Figure. Tabular representation for QUADAS-2 results

	RISK OF BIAS										APPLICABILITY CONCERNS			
**Study**	**Subject/model selection**		**Index test**			**Reference standard**		**Work flow**			**Subject/ model selection**	**Index test**		**Reference standard**
	**Q1**	**Q2**	**Q1**	**Q2**	**Q1**	**Q1**	**Q2**	**Q1**	**Q2**	**Q3**	**Q1**	**Q1**	**Q2**	**Q1**
Wong et al. (2005)	High	High	High	High	Unclear	Low	Low	Low	Unclear	Unclear	High	Low	High	Low
Hartmann et al. (2007)	High	High	High	Low	Low	Low	Low	Low	Low	High	High	Low	Low	Low
AlHadidi et al. (2011)	Unclear	High	Low	Low	Low	Low	Low	Low	Low	Low	Unclear	Low	Low	Low
Kim et al. (2011)	Low	High	High	Low	Low	Low	Low	Low	Low	High	Unclear	Low	Low	Low
Baek et al. (2012)	Low	High	Low	Low	Low	Low	Low	Low	Low	Low	Unclear	Unclear	Low	Low
Damstra et al. (2012)	Unclear	High	Low	Low	Low	Low	Low	Low	Low	High	Unclear	Low	Low	Low
Berssenbrügge et al. (2014)	High	High	Low	High	Low	Low	Low	Low	Unclear	Unclear	High	Low	High	Low
Wong et al. (2014)	Low	High	Low	High	Low	Low	Low	Low	Unclear	High	Low	Low	High	Low
Gateno et al. (2015)	High	High	High	Low	High	Unclear	High	High	High	High	High	High	High	Unclear
Kim et al. (2015)	Unclear	High	Low	Low	Low	Low	Low	Low	Unclear	Unclear	Unclear	Low	High	Low
Ryu et al. (2015)	Low	High	Low	Low	Low	Low	Low	Low	Low	Unclear	Low	Low	Low	Low
Lee et al. (2016)	Unclear	High	Low	Low	Low	Unclear	Low	Low	Unclear	Unclear	Unclear	Low	High	Unclear
Shin et al. (2016)	Low	High	Low	Low	Low	Low	Low	Low	Low	High	Unclear	Low	Low	Low
Song et al. (2016)	Low	High	Low	Low	Low	Low	Low	Low	Low	High	Low	Unclear	Low	Low
Sangln An et al. (2017)	High	High	Low	Low	Low	Low	Low	Low	Low	Unclear	High	Low	Low	Low
Dobai et al. (2018)	High	High	High	Low	Unclear	Unclear	Low	Low	Low	High	High	Low	Low	Unclear
Economou et al. (2018)	Unclear	High	Low	Low	Low	Low	Low	Low	Low	High	High	Low	Low	Low
Jajoo et al. (2018)	Low	High	High	Low	Unclear	Low	Low	Low	Unclear	High	Unclear	Low	High	Low
Oh et al. (2018)	Low	High	Low	Low	Low	Low	Low	Low	Low	Unclear	Low	Low	Low	Low
Thiesen et al. (2018)	Low	High	Low	Low	Low	Low	Low	Low	Low	High	Low	Low	Low	Low
Udomlarptham et al. (2018)	Low	High	Low	Low	Low	Low	Low	Low	Low	Unclear	Low	Low	Low	Low
Wong et al. (2018)	Unclear	High	Low	High	Low	Low	Unclear	Low	Low	High	Unclear	Low	Low	Low
Zhang et al. (2018)	Unclear	High	Low	Low	Low	Low	Low	Low	Low	Unclear	Unclear	Low	Low	Low
Zheng et al. (2018)	Unclear	High	Low	Low	Low	Unclear	Low	Low	Low	High	Unclear	Low	Low	Unclear

Choi et al. (2019)	Low	High	Unclear	Low	Unclear	Low	Low	Low	Low	Unclear	Unclear	Low	Low	Low
Kwon et al. (2019)	Low	High	Low	Low	Low	Low	Low	Low	Low	Unclear	Low	Low	Low	Low
Tan et al. (2019)	High	High	Unclear	Low	Unclear	Unclear	Low	Low	Unclear	Unclear	High	Low	High	Unclear
Vernucci et al. (2019)	Low	High	High	Low	Low	Low	Low	Low	Unclear	Unclear	Unclear	Low	High	Low
Han et al. (2020)	Low	High	Low	Low	Low	Low	Low	Low	Low	Unclear	Low	Low	Low	Low
Lee et al.(2020)	Low	High	Low	Low	Low	Low	Low	Low	Low	High	Low	Low	Low	Low
Ortún-Terrazas et al. (2020)	Unclear	High	Low	Low	Low	Low	Unclear	Low	Unclear	High	Unclear	Low	High	Low
Zhu et al. (2020)	Unclear	High	Low	Low	Unclear	Low	Low	Low	Unclear	High	Unclear	Low	High	Low
Jo et al. (2021)	Unclear	High	Low	Low	Low	Unclear	Low	Low	Low	High	Unclear	Unclear	Low	Unclear
Lv et al. (2021)	Low	Low	Low	Low	Low	Low	Low	Low	Low	Unclear	Low	Low	Low	Low
Mangal et al. (2021)	Low	High	Low	Low	Low	Low	Low	Low	Low	Unclear	Low	Low	Low	Low
Teng et al. (2021)	Unclear	Low	Low	Low	Low	Unclear	Low	Low	Unclear	Unclear	Unclear	Low	High	Unclear
Teng et al. (2021)	Low	Low	Low	Low	Low	Low	Low	Low	Low	Unclear	Low	Low	Low	Low
Ajmera et al. (2022)	Low	High	Low	Low	Low	Low	Low	Low	Low	Unclear	Low	Low	Low	Low
Feng et al. (2022)	Unclear	High	Low	Low	Low	Unclear	Low	Low	Low	Unclear	Unclear	Low	Low	Unclear
Grissom et al. (2022)	Low	Low	Low	Low	Unclear	Unclear	Low	Low	Low	High	Low	Low	Low	Unclear
Hsiao et al. (2022)	Unclear	High	Unclear	Low	Unclear	Low	Low	Low	Unclear	High	Unclear	Low	High	Low
Ajmera et al. (2023)	Low	High	Low	Low	Low	Low	Low	Low	Low	Low	Low	Low	Low	Low
